# Cardiorespiratory Events Following the Second Routine Immunization in Preterm Infants: Risk Assessment and Monitoring Recommendations

**DOI:** 10.3390/vaccines9080909

**Published:** 2021-08-16

**Authors:** Bettina Bohnhorst, Cornelia Weidlich, Corinna Peter, Carolin Böhne, Evelyn Kattner, Sabine Pirr

**Affiliations:** 1 Department of Pediatric Pneumology, Allergology and Neonatology, Hannover Medical School, 30625 Hannover, Lower Saxony, Germany; bohnhorst.bettina@mh-hannover.de (B.B.); weidlich.cornelia@mh-hannover.de (C.W.); peter.corinna@mh-hannover.de (C.P.); boehne.carolin@mh-hannover.de (C.B.); 2 Department of Neonatology, Children’s Hospital “Auf der Bult”, 30173 Hannover, Lower Saxony, Germany; ekattner@t-online.de

**Keywords:** preterm infant, routine immunization, second vaccination, cardiorespiratory events, monitoring

## Abstract

Due to frequent cardiorespiratory events (CREs) in response to the first routine immunization (rIM), current guidelines recommend readmitting and monitoring extremely preterm infants after the second rIM, though evidence on CREs in response to the second rIM is weak. In a prospective observational study, preterm infants with an increase in CREs after the first rIM were monitored for CREs before and after the second rIM. Seventy-one infants with a median gestational age of 26.4 weeks and a median weight of 820 g at birth were investigated at a median postnatal age of 94 days. All but seven infants showed an increase in CREs after the second rIM. The frequency of hypoxemias (*p* < 0.0001), apneas (*p* = 0.0003) and cardiorespiratory events requiring tactile stimulation (CRE-ts) (*p* = 0.0034) increased significantly. The 25 infants (35%) presenting with CRE-ts were significantly more likely to have been continuously hospitalized since birth (*p* = 0.001) and to receive analeptic therapy at the first rIM (*p* = 0.002) or some kind of respiratory support at the first (*p* = 0.005) and second rIM (*p* < 0.0001). At a postmenstruational age of 43.5 weeks, CRE-ts ceased. Our data support the recommendation to monitor infants who fulfil the above-mentioned criteria during the second rIM up to a postmenstruational age of 44 weeks.

## 1. Introduction

The current guidelines in many countries, such as Germany, United Kingdom, Australia, United States of America and Canada, state that preterm infants should receive routine immunization (rIM) according to the recommended schedule at their chronological age, without correction for prematurity [1,2,3,4,5]. The safety and efficacy of the vaccines Infanrix hexa (DTPa-HBV-IPV/Hib (hexavalent), GlaxoSmithKlein, Brentford, London, UK) and Prevenar 13 (Pneumococcal polysaccharide conjugate vaccine (13-valent, adsorbed), Pfizer Europe MA EEIG, Bruxelles, Belgium) in preterm infants has been previously demonstrated by several studies [6,7,8]. Multiple studies documented an increase or recurrence of cardiorespiratory events (CREs) including bradycardias, apneas and hypoxemias in response to the first rIM, particularly in extremely preterm infants born before 28 completed weeks of gestation [9,10,11,12,13]. Accordingly, several guidelines were published to closely observe hospitalized preterm infants for CREs following the first rIM [5,14,15]. Furthermore, readmission and monitoring over 48 to 72 h following the second rIM is recommended for extremely preterm infants who presented with an increase or recurrence of CREs in response to the first rIM [5,16]. However, the evidence on the frequency and timing of CREs following the second rIM in preterm infants is scarce and exclusively based on retrospective data [17,18,19]. The present study investigates prospectively the frequency and timing of CREs in response to the second rIM in preterm infants that presented with an increase or recurrence of such events after the first rIM. Furthermore, we sought to identify risk factors for the occurrence of such events in order to provide practice guidelines for the risk-adjusted patient management in the context of the second rIM. 

## 2. Materials and Methods

### 2.1. Recruitment

Preterm infants were recruited on the neonatal intermediate care unit of Hannover Medical School and “Auf der Bult” Children’s Hospital, Hannover, Germany, over a 3.5-year period. Inclusion criteria comprised the necessity of in-hospital monitoring during the second rIM [16] and written informed consent by the parents. Preterm infants with a 50% increase in frequency of CREs measured by a routinely applied scoring system adopted from Poets CF [20] or a CRE that required a medical intervention (CPAP and/or nasal cannula and/or caffeine therapy and/or doxapram therapy) following the 1st rIM qualified for monitoring during the 2nd rIM. Infants with severe inborn malformations including heart defects as well as acute illnesses prohibiting a second immunization were excluded. All infants received Infanrix hexa (DTPa-HBV-IPV/Hib (hexavalent), GlaxoSmithKlein, Brentford, London, UK) and Prevenar 13 (Pneumococcal polysaccharide conjugate vaccine (13-valent, adsorbed), Pfizer Europe MA EEIG, Bruxelles, Belgium) in 2 shots. 

### 2.2. Data Acquisition and Analysis

Epidemiological and clinical data were processed anonymously in Excel (Excel version 2010, Microsoft Corporation, Washington, DC, USA). We used VitaGuard 3100^®^ monitoring (GETEMED^®^ AG, Teltow, Germany) with a scanning frequency of one per second for heart rate (HR), respiratory rate (RR) and oxygen saturation (SpO_2_) for data recording. An averaging time of 2 s was applied for HR and RR. For SpO_2_, the FastSat mode was selected to enable rapid tracking of SpO_2_ by minimizing the averaging to 2 to 4 s. The infants’ HR, RR and SpO_2_ were recorded simultaneously by ECG, thoracic breathing movements and pulse oximeter plethysmography for 6 h prior and 48 h after immunization. Blue Sensor BRS ECG electrodes (AMBU^®^ GmbH, Bad Nauheim, Germany) were applied to the thorax and an LNOP Neo-L SpO_2_ sensor (Masimo^®^ Europe Ltd., Puchheim, Germany) was fixed on one foot. Recordings were carried out simultaneously to conventional monitoring used on the ward. The nursing staff noted each feeding episode in a standardized protocol. Furthermore, clinical data, medication and the requirement for respiratory support at the time of the 1st rIM and the 2nd rIM were recorded. Respiratory support included supplemental oxygen via nasal cannula, high flow nasal cannula or continuous positive airway pressure (CPAP). None of the included infants required invasive mechanical ventilation at the time of the 1st or 2nd rIM. Babies were laid down in supine position. The data recordings were evaluated using VitaWin^®^ software version 3.3 (GETEMED^®^ AG, Teltow, Germany).

A number of parameters were retrieved from these recordings including the overall recording time and the duration and percentage of artefact-free recording time. Any artefactual intervals of at least 4 s, in which the plethysmographic curve was disturbed by physical activity, were excluded from any further analyses. We assessed the basal values for HR, RR and SpO_2_ during periods of regular breathing not less than 10 s apart from any apneas or sighs. A regular breathing pattern was present if the breathing movement signal was steady in amplitude and rate. Bradycardias below 80 beats per minute (bpm), hypoxemias below 80% and apneas lasting for at least 10 s were classified as relevant CREs. The frequency, duration and severity of bradycardias below 80 bpm and of desaturations below 80% were determined during artefact-free recording. In addition, we evaluated the frequency and duration of apneas lasting at least 10 s during artefact-free recording. Finally, we analyzed the number of cardiorespiratory events that required a tactile stimulation (CRE-ts) of the infant in order to recover. Tactile stimulation was started not before 20 s after onset only when the infant showed no signs of spontaneous recovery or at a heart rate below 60 bpm and a SpO_2_ below 60% for more than 10 s. Each episode was documented, discriminating whether it occurred in the 6 h before immunization or within the first or the second 24 h after immunization.

### 2.3. Statistical Analyses

Data were analyzed anonymously and tested for Gaussian distribution using the Shapiro–Wilk normality test. Paired or unpaired Student’s *t*-tests, Mann–Whitney U tests, one-way ANOVA with post hoc Bonferroni’s multiple comparison tests and regression analyses were carried out as indicated using SPSS^®^ (version 25; SAS Institute, Cary, NC, USA) and GraphPad Prism^®^ (version 5; GraphPad software, San Diego, CA, USA). A *p* < 0.05 was considered statistically significant. 

### 2.4. Ethics

The local Ethics Committee of Hannover Medical School approved the study and written informed consent was obtained from all parents/legal guardians.

## 3. Results

**Figure 1:**
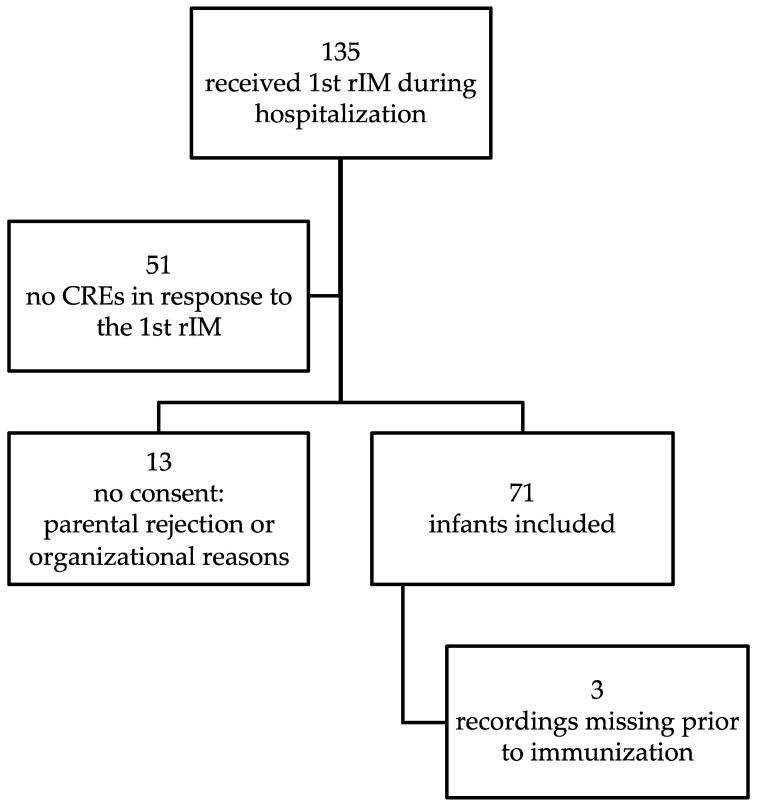
Flow chart of infant recruitment.

A total of 71 infants were included in the study, of which 61 were treated at Hannover Medical School and 10 at “Auf der Bult” Children’s Hospital, Hannover, Germany. For three infants, the recordings immediately prior to the immunization were missing due to organizational issues. Figure 1 provides detailed information on the patient recruitment process. Table 1 complements clinical data of included infants. Furthermore, four infants (5.6%) with a history of necrotizing enterocolitis of at least Bell stage IIb, one of whom required surgery, and 29 infants (40.8%) with a history of persistent ductus arteriosus requiring treatment were included in the study. None of the examined infants presented with a hemodynamically relevant persistent ductus arteriosus at the time of the second rIM. Fifty-one infants were born before 28 completed weeks of gestation, 20 infants had a gestational age (GA) of at least 28 weeks at birth. A total of 28 infants still required some kind of respiratory support at the time of the second rIM, 23 of these received supplemental oxygen via a nasal cannula, four infants required high flow nasal cannula and one infant CPAP at the time of the immunization. None of the infants received any analeptic therapy at the time of the second rIM.

The median total recording time was 55 h (range 40.5–66.9 h); the median recording time prior to immunization was 395 min (range 0–1399 min). A median of 71.8% (range 53.3–86.4%) of recording time was artefact-free. 

Median basal values for HR, RR and SpO_2_ were 143 bpm (range 121–173 bpm), 56 per minute (range 25–89 per minute) and 98% (range 91–100%), respectively, prior to immunization, and 147 bpm (range 120–169 bpm), 56 per minute (range 35–86 per minute) and 99% (range 93–100%), respectively, after immunization. No significant differences were detected between values for HR and RR prior to and after immunization. SpO_2_ values after immunization were significantly higher compared to values prior to immunization (*p* < 0.0001, paired *t*-test). This change was more significant in infants who did not require any respiratory support at the second rIM (*p* = 0.0001) than in infants who required respiratory support (*p* = 0.0126).

### CREs

Seven infants (9.9%) did not show an increase in CREs in response to the second rIM. Four of these did not present any CREs either prior to or after immunization; three infants showed a decreasing frequency of CREs. These infants were less likely to be continuously hospitalized until the second rIM (28.6% vs. 71.9%, *p* = 0.0217) and less frequently required pharmacological analeptic therapy at the first rIM (28.6% vs. 76.6%, *p* = 0.0171) compared to infants with an increased number of CREs after the second rIM. Other clinical parameters such as GA, weight, intraventricular hemorrhage or bronchopulmonary dysplasia did not differ significantly between both groups. 

Results on bradycardias, hypoxemias, apneas and CRE-ts are shown in Table 2. 

For a better evaluation of the clinical relevance of the CREs, Table 3 shows frequencies per hour only in those infants that presented with the respective CRE.

Three infants (4.4%) showed CRE-ts prior to immunization, of which two also presented such events after immunization. All three infants were continuously hospitalized until the second rIM. Four of the 71 infants included in this analysis (5.6%) did not show any CRE-ts prior to or during the first 24 h after immunization; however, these infants presented with such events during the second 24 h after immunization. Of these, three infants showed one event each; one infant showed two such events. All four infants were continuously hospitalized until the second rIM.

In three infants, supplemental oxygen via nasal cannula was newly initiated due to repeated CRE-ts after the second rIM. No infant required bag-mask ventilation or initiation of mechanical ventilatory support such as high flow nasal cannula, CPAP, or intubation.

**Figure 2:**
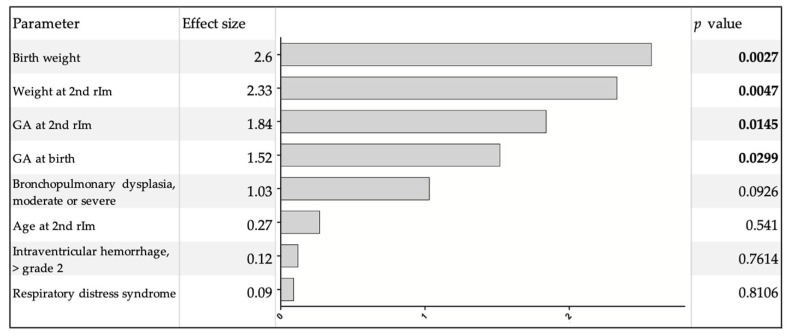
Multivariate regression analysis of possible risk factors for CRE-ts. Significant values are shown in bold.

Clinical characteristics of infants who presented with CRE-ts after immunization compared to infants without such events are shown in Table 4. All infants who later presented with CRE-ts in response to the second rIM required either respiratory support or analeptic treatment at the time of the first rIM. All but two infants who presented with CRE-ts were continuously hospitalized until the second rIM. Both infants who were discharged and readmitted for their second rIM showed CRE-ts during the first 24 h after immunization. One of them presented an additional event in the second 24 h after immunization. Multivariate regression analysis revealed that solely GA and weight both at birth and at the second rIM were significantly associated with the occurrence of CRE-ts. Morbidities potentially associated with respiratory instability in this patient cohort as bronchopulmonary dysplasia, intraventricular hemorrhage or respiratory distress syndrome did not show this association (Figure 2). Linear regression analysis showed that CRE-ts in response to the second rIM cease at a GA of more than 43.5 weeks (*p* = 0.0164, r^2^ = 0.0806).

## 4. Discussion

We prospectively analyzed the occurrence of CREs in response to the second rIM in extremely preterm infants at the chronological age of approximately 3 months, which roughly correlates with their expected delivery date.

Baseline values for HR, RR and SpO_2_ were in line with values reported for preterm infants of similar postmenstruational age (PMA) [21,22,23,24,25,26,27]. Compared to healthy term newborns preterm infants in our study showed a higher baseline for HR, RR but similar values for SpO_2_ [25,28]. Our data confirm that HR and RR decrease with increasing GA and PMA [29,30].

HR and RR did not change significantly in response to immunization. However, the SpO_2_ was significantly but only slightly higher thereafter (98% before vs. 99% after immunization). Interestingly, this difference, while clinically hardly relevant, was more pronounced in infants who did not require any respiratory support at the time of the second rIM in comparison to infants who required respiratory support. Therefore, this increase does not represent an effect of supplemental oxygen therapy. Instead, we speculate that the inflammatory response to the immunization might cause vasodilatation, thus augmenting peripheral perfusion and oxygen transport. 

In clinical practice, the most common definition of bradycardia is a drop of HR to less than 80 bpm. Studies have shown that in preterm neonates, cerebral perfusion is significantly reduced below this cut-off value [31]. Hypoxemia is most commonly defined as a drop of SpO_2_ below 80%. These definitions are also applied by the German guidelines on the therapy of idiopathic apnea, bradycardia and hypoxemia [32]. The frequency, duration and severity of bradycardias and hypoxemias recorded prior to immunization were similar to values previously reported in preterm infants of similar PMA [24,26,27]. Compared to healthy term newborns, the overall number of preterm infants in our study presenting with hypoxemias was increased (28% vs. 72.1%). However, the number of hypoxemias per hour was within the range reported in term neonates [28]. Data on the frequency of apneas vary noticeably between different studies, with median values of 1.7 to 16.1 per hour and a proportion of infants up to 98% in healthy term neonates [28,33,34,35]. We found apneas in only 26.5% of infants with a frequency of 0.1 per hour for all infants and 0.4 per hour exclusively in infants presenting with apneas. However, in the current literature, different definitions of apnea with a range of 3 to 10 s were applied [28,33,34,35]. In contrast, we chose to only include apneas of at least 10 s, thus representing a higher clinical significance. Overall, bradycardias occurred rarely (0.1/hour), but still more frequently in our preterm patients compared to healthy term neonates [28,36].

In response to immunization, the number of hypoxemias, apneas and CRE-ts per hour increased significantly during the first 24 h after the procedure. Within the second 24 h after immunization, the frequency of these events decreased significantly and returned to baseline values. 

The incidence of CREs associated with the first rIM varies widely in the available literature [9,10,11,13,37,38]. This might be due to the various definitions for such events and differing methods of monitoring. Unsurprisingly, in our study, the number of infants presenting with CREs in response to second rIM and the severity of such events were lower compared to studies investigating CREs in connection with the first rIM [11,13,37,38]. Most likely, this can be explained by the higher PMA of infants at the second rIM as the incidence of CREs in preterm infants is inversely related to GA and PMA [36,39,40]. 

Compared to previous studies exploring CREs in response to the second rIM in preterm infants, we found an increase of such events in our patients [17,18,19]. However, the above-mentioned studies retrospectively evaluated routine in-patient monitoring and nursing staff documentation for CREs. The latter is known to be unreliable and may miss many events compared to monitor recordings [41,42]. This leads to an underestimation of actual events and might explain the increase of CREs in our study. Furthermore, immunization recommendations differ in varying countries: Australia schedules the second rIM at the fourth month of age, in contrast to the third month as recommended in Canada and most European countries including Germany and the United Kingdom [1,2,3,5]. The increased PMA at the time of the second rIM is probably responsible for the reduced incidence of CREs in Australian studies [18,19]. 

The observation of significantly decreased CREs during the second 24 h after immunization would support the notion that monitoring after this procedure can be kept to a maximum of 24 h. However, four of the included infants showed CRE-ts exclusively within the second 24 h after immunization. These would have been missed if monitoring was cut short. Therefore, a general recommendation to shorten the monitoring to 24 h cannot be supported by our data. However, infants who presented with CRE-ts were of significantly lower GA and weight at birth and at the second rIM, respectively. Furthermore, the need for analeptic therapy at the first rIM and for respiratory support at the first and second rIM was increased. In fact, all infants who presented with CRE-ts at the second rIM required either respiratory support or analeptic therapy or both at the first rIM. The presence of any of the following complications of prematurity, bronchopulmonary dysplasia, intraventricular hemorrhage or respiratory distress syndrome was not associated with the occurrence of CRE-ts. On the contrary, factors related to the maturity of the infant as weight and GA determine the risk of CRE-ts in response to the second rIM. 

**Figure 3:**
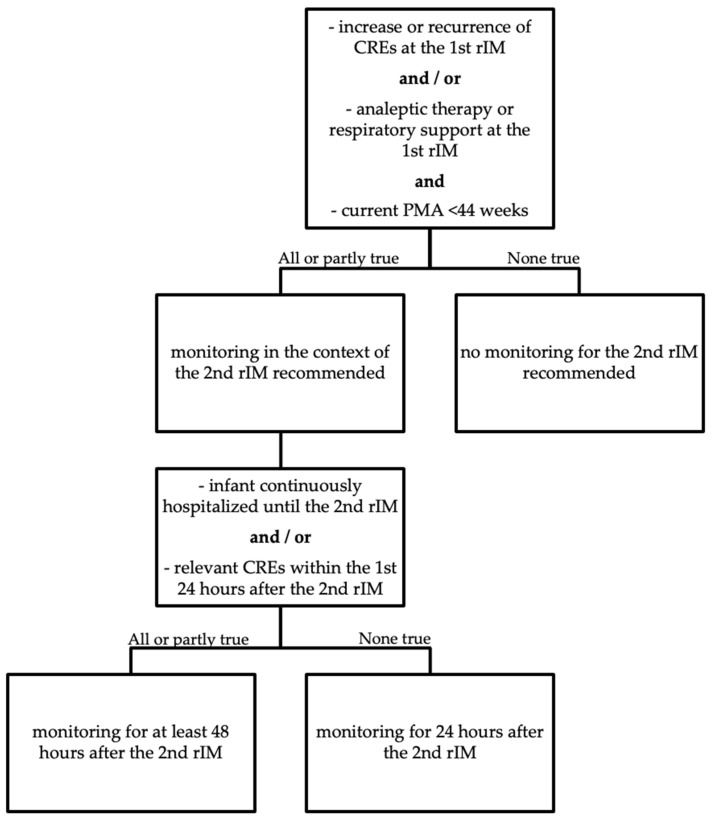
Flow chart of monitoring recommendations for preterm infants in the context of the 2nd rIM.

All infants who were discharged prior to the second rIM either did not show any CRE-ts at all or presented with such events already within the first 24 h after immunization. Therefore, in case of an uneventful monitoring during the first 24 h, it seems safe in those patients to discontinue monitoring and discharge these infants already 24 h post-vaccination. In contrast, infants presenting with relevant CREs during the first 24 h or who were still hospitalized prior to the second rIM should be monitored for at least 48 h after immunization. Figure 3 summarizes these recommendations in a flow chart for easy decision-making.

Regression analyses defined a PMA of 43.5 weeks at the time of the second rIM as the age at which immunization-related CRE-ts will cease. This is consistent with the reported age of approximately 43 weeks PMA at which the frequency of CREs in preterm infants has decreased to the level observed in term born infants [36]. This finding favors the reduced schedule in preterm infants with the second rIM given at a chronological age of 4 months as applied in the United States of America and Australia [3,4] and implemented for term infants in Germany and Canada (2 + 1 schedule) [1,5]. However, most efficacy studies of immunization in preterm infants so far have been based on a three-dose primary vaccination (3 + 1 schedule) [7,43,44]. Further studies are needed to adopt a 2 + 1 schedule for preterm infants. 

To our knowledge, this is the first prospective study to analyze CREs in response to the second rIM in preterm infants and to provide evidenced guidance for the monitoring of these infants with regard to the second rIM. Limitations are the restriction to only two study sites and one kind of vaccine combination which limits generalizability. Furthermore, as the recordings were limited to 48 h after the immunization, no statement on any further CREs and the need for an extended monitoring thereafter can be made.

## 5. Conclusions

In conclusion, most preterm infants who showed an increase or recurrence of CREs in response to the first rIM also presented with increased hypoxemias, apneas and CRE-ts after the second rIM at a chronological age of 3 months. As risk factors for an overall increase of CREs and persistence for more than 24 h after immunization, we identified low GA and weight at birth and at the time of the second rIM, the need for analeptic therapy and respiratory support at the first rIM and continuous hospitalization until the second rIM. CRE-ts ceased at a PMA of 43.5 weeks. Consequently, we recommend monitoring in the context of the second rIM for infants with a current PMA of less than 44 weeks who presented either with an increase or recurrence of CREs or the need for analeptic therapy or respiratory support at the first rIM for a minimum of 24 h. Monitoring should be extended to a minimum of 48 h for infants who were still hospitalized at the time of the second rIM or showed relevant CREs within the first 24 h after immunization.

## Figures and Tables

**Table 1:** Clinical data of included infants.

Clinical Characteristics	Total *n* = 71
GA at birth in weeks (median (range))	26.4 (22.7–30.9)
Birth weight in grams (median (range))	820 (480–1460)
Age at 2nd rIM in days (median (range))	94 (85–121)
GA at 2nd rIM in weeks (median (range))	40.0 (36.3–44.7)
Weight at 2nd rIM in grams (median (range))	2900 (1890–4080)
Analeptic treatment at 1st rIM (*n* (%))	49 (69.0)
Respiratory support at 1st rIM (*n* (%))	47 (66.2)
Respiratory support at 2nd rIM (*n* (%))	28 (39.4)
Bronchopulmonary dysplasia, moderate or severe (*n* (%))	22 (31.0)
Intraventricular hemorrhage, >grade 2 (*n* (%))	10 (14.1)

**Table 2:** Results on bradycardias, hypoxemias, apneas and CRE-ts for all included infants.

	6 h Pre-Immunization *n* = 68	0–24 h Post-Immunization *n* = 71	24–48 h Post-Immunization *n* = 71	*p* Value Pre- vs. Post-Immunization ^a^	*p* Value 0–24 vs. 24–48 h Post-Immunization ^b^
**Bradycardias**					
Number of infants, *n* (%)	16 (23.5)	38 (53.5)	34 (47.9)	**<0.0001**	n.s.
Number per hour, mean (SD)	0.1 (0.2)	0.15 (0.4)	0.1 (0.2)	0.1023	n.s.
Lowest HR (bpm), mean (SD)	68 (10)	65 (10)	64 (11)	0.4185	n.s.
Maximum duration (seconds), mean (SD)	6.1 (5.1)	12.4 (28.5)	10.3 (9.9)	0.5737	n.s.
**Hypoxemias**					
Number of infants, *n* (%)	49 (72.1)	67 (94.4)	61 (85.9)	**0.0002**	n.s.
Number per hour, mean (SD)	1.6 (3.1)	4.0 (7.8)	1.7 (3.1)	**<0.0001**	**0.0004**
Lowest SpO_2_ (%), mean (SD)	64 (15)	52 (21)	57 (16)	**0.0023**	n.s.
Maximum duration (seconds), mean (SD)	18.5 (30.4)	33.5 (65.1)	21.3 (21.6)	0.1506	n.s.
**Apneas**					
Number of infants, *n* (%)	18 (26.5)	44 (62.0)	32 (45.1)	**<0.0001**	**0.0320**
Number per hour, mean (SD)	0.1 (0.3)	0.3 (0.8)	0.1 (0.2)	**0.0030**	**0.0076**
Maximum duration (seconds), mean (SD)	10.9 (1.9)	14.6 (7.3)	13.5 (6.0)	0.1124	n.s.
**CRE-ts**					
Number of infants, *n* (%)	3 (4.4)	21 (29.6)	12 (16.9)	**<0.0001**	**0.0280**
Number per hour, mean (SD)	0.01 (0.03)	0.02 (0.05)	0.01 (0.03)	**0.0034**	**0.0222**

^a^ one-way ANOVA. ^b^ post hoc Bonferroni’s multiple comparison test, if significant a Student’s *t*-test was run. n.s., not significant. Significant values are shown in bold.

**Table 3:** Number of CREs per hour only in infants that presented with the respective CRE.

	6 h Pre-Immunization	0–24 h Post-Immunization	24–48 h Post-Immunization
**Bradycardias**			
Number of infants, *n*	16	38	34
Number per hour, mean (SD)	0.3 (0.3)	0.3 (0.5)	0.2 (0.3)
**Hypoxemias**			
Number of infants, *n*	49	67	61
Number per hour, mean (SD)	2.3 (3.5)	4.1 (7.8)	1.9 (3.2)
**Apneas**			
Number of infants, *n*	18	44	32
Number per hour, mean (SD)	0.4 (0.5)	0.5 (0.9)	0.2 (0.3)
**CRE-ts**			
Number of infants, *n*	3	21	12
Number per hour, mean (SD)	0.1 (0.1)	0.1 (0.1)	0.1 (0.04)

**Table 4:** Clinical data of infants with and without CRE-ts after the second immunization.

	Infants without CRE-ts *n* = 46	Infants with CRE-ts *n* = 25	*p* Value
GA at birth in weeks (median (range))	27.1 (23.6–30.9)	25.4 (22.7–29.3)	**0.0024** ^a^
Birth weight in grams (median (range))	927.5 (480–1460)	690 (520–1235)	**0.0022** ^a^
Age at 2nd rIM in days (median (range))	94 (88–121)	94 (85–96)	0.4483 ^b^
GA at 2nd rIM in weeks (median (range))	40.9 (36.7–44.7)	38.3 (36.3–42.6)	**0.0002** ^a^
Weight at 2nd rIM in grams (median (range))	3080 (1890–4080)	2680 (2035–3770)	**0.0026** ^a^
Analeptic treatment at 1st rIM (*n* (%))	26 (56.5)	23 (92.0)	**0.0022** ^b^
Respiratory support at 1st rIM (*n* (%))	25 (54.3)	22 (88.0)	**0.0046** ^b^
Respiratory support at 2nd rIM (*n* (%))	10 (21.7)	18 (72.0)	**<0.0001** ^b^
Discharge home and readmission for 2nd rIM (*n* (%))	21 (45.7)	2 (8.0)	**0.0013** ^b^
Bronchopulmonary dysplasia, moderate or severe (*n* (%))	12 (26.1)	10 (40.0)	0.2322 ^b^
Intraventricular hemorrhage, >grade 2 (*n* (%))	4 (8.7)	6 (24.0)	0.0804 ^b^

^a^ unpaired Student’s *t*-test. ^b^ Mann–Whitney U test. Significant values are shown in bold.

## Data Availability

The data presented in this study are available on justified request from the corresponding author. The data are not publicly available due to privacy restrictions. Public data sharing is not in accordance with the consent provided by the participants on the use of confidential data.
